# Supplementary material for: The Eyelid Angiosarcoma: A Systematic Review of Characteristics and Clinical Course
Source: J Clin Med. 2022 Jul 20;11(14):4204. doi: 10.3390/jcm11144204 (PMC9320659; doi:10.3390/jcm11144204)
Supplement: Supplementary file 1 [file jcm-11-04204-s001.zip › Supplementary Figures S1 and S2.pdf]

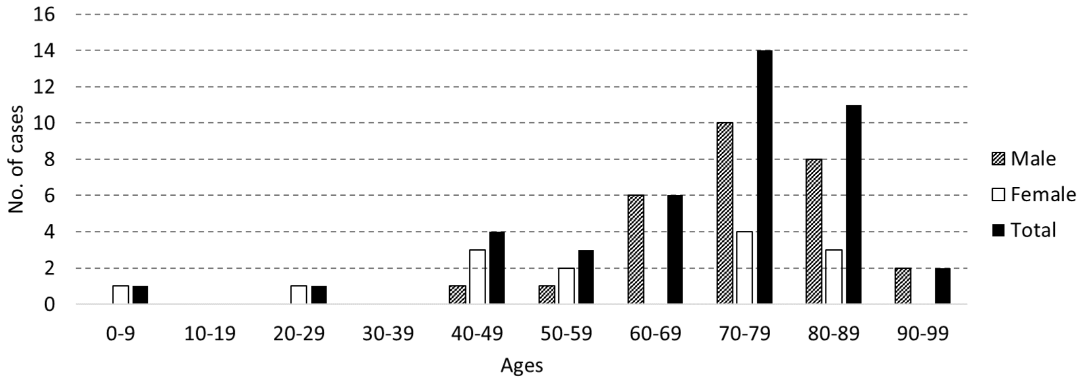


**Figure S1**. Age and sex distribution of eyelid angiosarcoma.

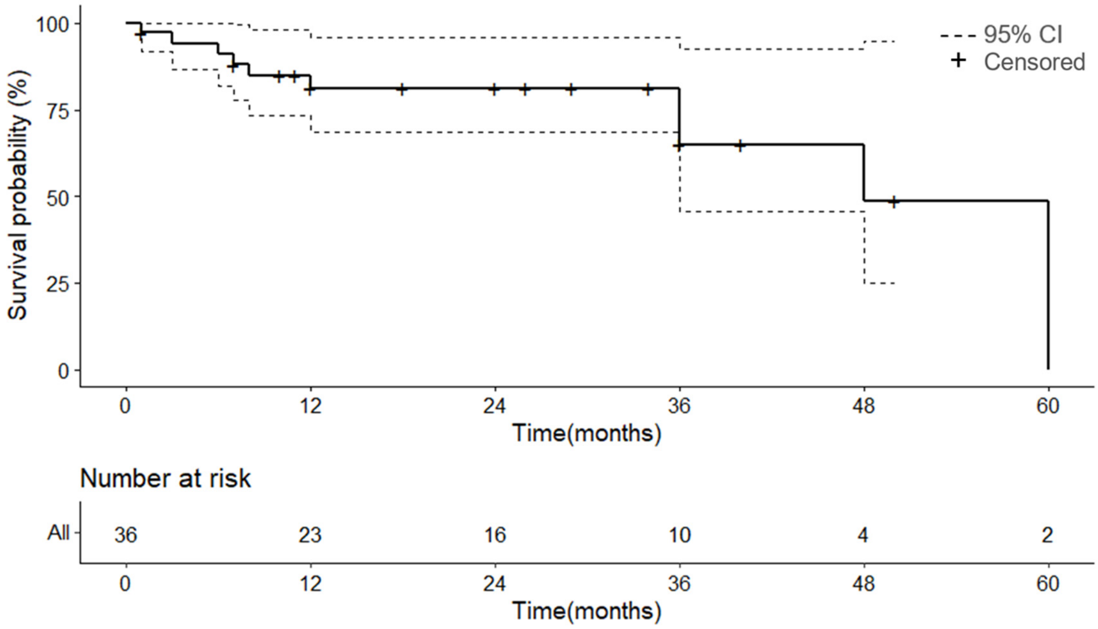


**Figure S2**. Survival probability of patients with eyelid angiosarcoma.
