# Supplementary material for: The Eyelid Angiosarcoma: A Systematic Review of Characteristics and Clinical Course
Source: J Clin Med. 2022 Jul 20;11(14):4204. doi: 10.3390/jcm11144204 (PMC9320659; doi:10.3390/jcm11144204)
Supplement: Supplementary file 1 [file jcm-11-04204-s001.zip › Supplementary Table S2.pdf]

Supplementary Table S2. Critical appraisal checklist of case reports included in the analysis.

Study	Demographic Characteristics*	Clinical History	Clinical Condition on Presentation	Diagnostic Tests	Treat ment	Post Intervention Conditions	Adverse Events	Takeaway Lessons
De Schweinitz [5]	Yes	Yes	Yes	Yes	Yes	Yes	No	Yes
Mackenzie [6]	Unclear	Yes	Yes	Yes	Yes	Yes	No	Yes
Panizzon [7]	Yes	Yes	Yes	Yes	Yes	Yes	No	Yes
Bray [8]	Yes	Yes	Yes	Yes	Yes	Yes	No	Yes
Lapidus [9]	Unclear	Yes	Yes	Yes	Yes	Yes	No	No
Gunduz [10]	Unclear	Yes	Yes	Yes	Yes	Yes	No	Yes
Mehrens [11]	Unclear	Yes	Yes	Yes	Yes	Yes	No	Yes
Tay [12]	Yes	Yes	Yes	Yes	Yes	Yes	No	Yes
Conway [13]	Yes	Yes	Yes	Yes	Yes	Yes	No	Yes
Hiemstra [14]	Yes	Yes	Yes	Yes	Yes	Yes	No	Yes
Kikuchi [15]	Yes	Yes	Yes	Yes	Yes	Yes	No	Yes
Miura [16]	Yes	Yes	Yes	Yes	Yes	No	No	Yes
De Keizer [17]	Unclear	Yes	Yes	Yes	Yes	Yes	No	Yes
Mitra [18]	Yes	Yes	Yes	Yes	Yes	Yes	No	Yes
Sluzevich [19]	Yes	Yes	Yes	Yes	Yes	Yes	No	Yes
Fuller [20]	No	Yes	Yes	Yes	Yes	Yes	No	Yes
Papalas [21]	Yes	Yes	Yes	Yes	Yes	Yes	No	Yes
Wiwatwongwana [22]	Yes	Yes	Yes	Yes	Yes	Yes	No	Yes
Lee [23]	Yes	Yes	Yes	Yes	Yes	No	No	Yes
Ambujam [24]	Unclear	Yes	Yes	Yes	Yes	No	No	Yes
Choi [25]	Yes	Yes	Yes	Yes	Yes	Yes	No	Yes
Cox [26]	Unclear	Yes	Yes	Yes	Yes	Yes	No	Yes
Demirci [27]	Unclear	Yes	Yes	Yes	Yes	Yes	No	Yes
Karkouche [28]	Unclear	Yes	Yes	Yes	Yes	Yes	No	Yes
Dempsey [29]	Yes	Yes	Yes	Yes	Yes	Yes	No	Yes
Kim [30]	Yes	Yes	Yes	Yes	Yes	No	No	Yes
Lemanski [31]	Unclear	Yes	Yes	Yes	Yes	Yes	No	Yes
Benmously [32]	Unclear	Yes	Yes	Yes	Yes	Yes	No	Yes
Huang [33]	Unclear	Yes	Yes	Yes	Yes	Yes	No	Yes
Hamill [34]	Unclear	Yes	Yes	Yes	Yes	No	No	Yes
Ferguson [35]	Unclear	Yes	Yes	Yes	Yes	Yes	No	Yes
Milman [36]	Yes	Yes	Yes	Yes	Yes	No	No	Yes

*Demographic characteristics includes age, sex and race.
